# The role of alpha oscillations in free‐ and goal‐directed semantic associations

**DOI:** 10.1002/hbm.26770

**Published:** 2024-07-05

**Authors:** Ioanna Zioga, Yoed N. Kenett, Anastasios Giannopoulos, Caroline Di Bernardi Luft

**Affiliations:** ^1^ Donders Institute for Brain, Cognition and Behaviour Radboud University Nijmegen The Netherlands; ^2^ Faculty of Data and Decision Sciences, Technion—Israel Institute of Technology Haifa Israel; ^3^ School of Electrical and Computer Engineering National Technical University of Athens (NTUA) Athens Athens Greece; ^4^ Division of Psychology, CHMLS—Life Sciences Brunel University London London UK

**Keywords:** alpha oscillations, creativity, EEG, free associations, goal‐directed, instructions, semantic associations, semantic cognition, semantic control

## Abstract

Alpha oscillations are known to play a central role in several higher‐order cognitive functions, especially selective attention, working memory, semantic memory, and creative thinking. Nonetheless, we still know very little about the role of alpha in the generation of more remote semantic associations, which is key to creative and semantic cognition. Furthermore, it remains unclear how these oscillations are shaped by the intention to “be creative,” which is the case in most creativity tasks. We aimed to address these gaps in two experiments. In Experiment 1, we compared alpha oscillatory activity (using a method which distinguishes genuine oscillatory activity from transient events) during the generation of free associations which were more vs. less distant from a given concept. In Experiment 2, we replicated these findings and also compared alpha oscillatory activity when people were generating free associations versus associations with the instruction to be creative (i.e. goal‐directed). We found that alpha was consistently higher during the generation of more distant semantic associations, in both experiments. This effect was widespread, involving areas in both left and right hemispheres. Importantly, the instruction to be creative seems to increase alpha phase synchronisation from left to right temporal brain areas, suggesting that intention to be creative changed the flux of information in the brain, likely reflecting an increase in top‐down control of semantic search processes. We conclude that goal‐directed generation of remote associations relies on top‐down mechanisms compared to when associations are freely generated.

## Graphical abstract

We measured alpha oscillations during the generation associations (a) in free and goal‐directed semantic tasks. We observed higher alpha (b) during the generation of remote‐free associations. However, alpha was higher during goal‐directed associations (c). Alpha synchronised from the left to the right temporal areas during goal‐directed associations (d).

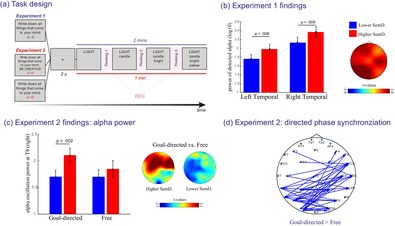

## 1 INTRODUCTION

Alpha was the first electroencephalographic (EEG) rhythm to be observed in the human EEG (Adrian & Matthews, 1934). Since it was first observed by Hans Berger in 1929, research on alpha brain oscillations has evolved substantially, especially regarding our understanding of its role on brain operations and cognitive functions. There is evidence that event‐related increases in alpha power, also called event‐related synchronisation (ERS), represent inhibitory processes associated with precisely timed suppression of neural firing (Haegens, Händel, & Jensen, 2011; Haegens, Nácher, et al., 2011). One of the explanations for this suppression is that it represents an active inhibition of task‐irrelevant areas thus fine‐tuning sensory processing (Jensen & Mazaheri, 2010; Klimesch, 2012; Klimesch et al., 2007). Increase in alpha oscillatory activity has been observed in a number of cognitive tasks, especially tasks which rely on executive control such as attention (Haegens, Händel, & Jensen, 2011; Wöstmann et al., 2019) and working memory (Jensen et al., 2002), as well as higher‐level complex tasks, such as language comprehension (Zioga et al., 2023). For instance, it was found that the higher the number of items to be remembered, the higher the alpha power in the upper frequency range (>9 Hz) during retention (Jensen et al., 2002; Scheeringa et al., 2009; Tuladhar et al., 2007). ERS in alpha was found to be a neural signature of distractor suppression independently of the attentional target (Wöstmann et al., 2019). Thus, Wöstmann et al. (2019) suggested that these alpha oscillations depend directly on the participant's intention to ignore distracting information.

Beyond these executive functions, alpha ERS has also been consistently observed in several aspects of the creative process (e.g., Agnoli et al., 2020; Benedek et al., 2011; Camarda et al., 2018; Eymann et al., 2022; Fink & Benedek, 2014; Fink & Neubauer, 2006; Luft et al., 2018; Mastria et al., 2021; Perchtold‐Stefan et al., 2020, 2023; Sandkühler & Bhattacharya, 2008; Stevens Jr & Zabelina, 2019; Yu et al., 2023). Notwithstanding the consistency of the involvement of alpha oscillations in creativity tasks, the topography and characteristics of alpha oscillatory activity during creativity tasks vary considerably. As an attempt to address this issue, Luft et al. (2018) conducted a series of experiments to test the hypothesis that alpha oscillations in the right temporal lobe are involved in inhibiting obvious or closer semantic associations, which often get in the way when people try to come up with more remote or creative ideas. Considering the key role of the right temporal area on drawing semantic associations (Binder et al., 2009; Jung‐Beeman, 2005; Lambon Ralph et al., 2010; St George et al., 1999; Tranel et al., 1997), previous findings (Luft et al., 2018) indicated that inhibiting obvious ideas depend on alpha oscillatory activity in a task‐relevant area. Based on these findings, it was suggested that right temporal alpha oscillations in creative tasks represent the active process of inhibiting obvious associations when attempting to come up with more creative ideas, which is necessary for selective access to knowledge systems (Klimesch, 2012).

Here we ask whether higher right temporal alpha oscillations during idea generation could be associated with the generation of more semantically distant associations in free and goal‐directed association tasks, which have both been implicated in the creative process (Beaty & Kenett, 2023). Beaty and Kenett (2023) have recently reviewed the role of associative abilities as a general mechanism in creative thinking. Specifically, they distinguished between free‐, unintentional, and goal‐directed, intentional, associations. Specifically, the authors argue that higher creative individuals: (1) travel “further” in semantic memory when associating (Beaty et al., 2021; Gray et al., 2019), (2) switch between more semantic subcategories (Zhang et al., 2023), and (3) make larger leaps between associations (Olson et al., 2021).

Free association tasks have been validated as a measure of creative thinking and are increasingly used as a valid measure of creativity (Beaty et al., 2021; Benedek et al., 2012; Gray et al., 2019). In free association tasks, participants are presented with a word and told to come up with semantic associations freely, just reporting what spontaneously comes to mind in a chain. Despite the popularity of such tasks to measure creativity, we know very little about the neural correlates of producing semantic associations in these tasks and how different they are when we perform intentional, goal‐directed associations. We address this gap in the literature by comparing alpha oscillatory activity during the generation of closer and more distant semantic associations in a free association task (Experiment 1).

To understand free associations, it is fundamental to analyse how they differ from goa‐directed associations. For instance, there is an important difference between free association and traditional creativity tasks. Most creativity tasks (divergent and convergent) require participants to be creative, even when creativity is not explicitly mentioned in the instructions (e.g., coming up with unusual uses for objects, story titles, solving a problem whose solution relies on creative thinking, etc.). On the other hand, in free association tasks, participants are told to express what comes to their mind first, or spontaneously. Therefore, the nature of a free association task is in essence different from typical creativity tasks, especially regarding its goal‐directed aspect. Furthermore, previous studies on creativity have shown (Chen et al., 2005; Harrington, 1975; Niu & Liu, 2009; Nusbaum et al., 2014) that instructing participants to be creative does result in more creative outputs. More than a mere instruction, telling participants to be creative might change the pattern of responses (Kaya & Acar, 2019) and the actual neural mechanisms via which creative responses are achieved, as suggested by Nusbaum et al. (2014). For instance, a previous study (Nusbaum et al., 2014) observed that when instructed to be creative, participants with higher levels of intelligence produced disproportionally more creative responses. This effect could be due to a potentially stronger involvement of top‐down processes when participants are told to be creative, an instruction which tends to be more effective in participants with higher intelligence. We address this gap in Experiment 2 by comparing alpha oscillatory activity during the generation of free‐ versus goal‐directed associations.

The effect of instruction was also tested in semantic association tasks (Heinen & Johnson, 2018). Heinen and Johnson (2018) demonstrated that when participants were cued to be creative by generating novel and appropriate responses, they generated associations with higher semantic distances than when instructed to generate random associations (novel but not appropriate). Notwithstanding the robust findings regarding the instruction to be creative and the hypothetical differences in the mechanisms behind it, we still do not know if the brain processes behind intentional idea generation are different from processes of spontaneous free associations, which have been used to measure creativity. Understanding such differences is also very important when considering the processes behind different measures of creativity which, on surface, look similar, intend to measure a similarly defined construct, and are moderately correlated. For instance, a recently developed measure of creativity via semantic associations, the Divergent Association Task (Olson et al., 2021), requires participants to generate goal‐directed distant or remote associations, while free association task requires the participant to state what comes to mind first, which is a more spontaneous process. Therefore, having a better understanding of the different processes behind free‐ versus goal‐directed creative associations will be key to interpreting the findings obtained using these distinct measures.

Despite the abundance of studies looking at brain oscillations during creativity tasks (e.g., Agnoli et al., 2020; Benedek et al., 2011; Camarda et al., 2018; Eymann et al., 2022; Fink & Neubauer, 2006; Luft et al., 2018; Mastria et al., 2021; Perchtold‐Stefan et al., 2020; Sandkühler & Bhattacharya, 2008; Yu et al., 2023), there has been no research (to the best of our knowledge) looking at alpha oscillatory processes during the generation of semantic associations in free association tasks, despite its widespread use in behavioural studies as a creativity measure. Additionally, most studies looking at alpha oscillatory activity during creative thinking use standard methods based on band pass filtering (e.g., FFT, power spectral density), which include both aperiodic and periodic components of the EEG signal. The aperiodic 1/f signal follows a power law distribution and reflects the non‐oscillatory activity present in the EEG power spectrum, whereas the periodic component reflects the oscillatory activity of interest (Gerster et al., 2022). The presence of genuine brain oscillations is best verified by identifying peaks in the spectrum with power over and above the aperiodic 1/f signal (Donoghue et al., 2020). For this purpose, we used the Better OSCillation Detection (BOSC) method (Hughes et al., 2012; Seymour et al., 2022; Whitten et al., 2011) which disentangles arrhythmical background 1/f activity from the periodic signal. In Experiment 1, we compare the alpha brain oscillations during the generation of free associations of higher (distant) versus lower (close) semantic distances. We expect alpha to be higher at the right temporal area during the generation of more distant semantic associations. Here we focused on the right temporal area since there is evidence that alpha oscillatory activity in this region is involved in creative thinking (Agnoli et al., 2020; Camarda et al., 2018; Luft et al., 2018). Previous work from our group provided evidence that alpha oscillatory activity in this region represents the active inhibition of obvious or close semantic associations (Luft et al., 2018). Therefore, we expect that this region, as opposed to the left temporal area (control region), would present higher alpha oscillatory activity during the generation of more distant semantic associations, especially in the goal‐directed task.

In Experiment 2, we aim to examine how the intention to be creative may affect alpha oscillatory activity. We address this question by investigating alpha oscillations during the generation of free associations compared to goal‐directed associations in which participants are told to “be creative” and inhibit the first ideas that come to mind. We hypothesise that if right temporal alpha represents the inhibition of obvious ideas, it will be higher during goal‐directed creative associations. Furthermore, our hypothesis that the inhibitory nature of this process is “active” and “directed” towards semantic search processes requires that we evaluate the effective connectivity patterns during the generation of associations under these two conditions. We estimated effective connectivity using a measure called the phase slope index (PSI), which uses the phase slope of the EEG signals to estimate the direction of the information flux in specific frequency bands (Nolte et al., 2008). Considering that cortical alpha oscillations are likely to flow from higher‐ to lower‐order areas and likely to represent top‐down processes (Halgren et al., 2019), we expected that right temporal alpha oscillations would be driven from higher‐order areas (left frontal and parietal) during intentionally creative semantic associations. We address this question in Experiment 2 by investigating directed phase synchronisation in the alpha band during free compared to goal‐directed associations. Since there are no previous studies looking at effective connectivity during the generation of semantic associations, we used a data‐driven method (non‐parametric cluster permutation) to understand the differences in directed phase synchronisation between different brain regions in the alpha frequency band.

## 2 EXPERIMENT 1

First, we investigated the differences in alpha oscillatory activity when participants are required to come up with associations of lower versus higher semantic distance during a free association task.

### 2.1 Methods

#### 2.1.1 Participants

One hundred and thirty healthy adults (67 female) aged between 18 and 32 years old (21.80 ± 2.63 years, mean ± SD) took part in the experiment. We included in the analysis only participants who gave at least 10 responses in the free association task and therefore excluded 30 participants, resulting in a total number of 100 participants. One participant was excluded due to noisy data. A further 20 participants were excluded due to insufficient EEG data. The final dataset analysed had 79 participants in total (36 female, age 22.11 ± 2.79 years, mean ± SD). All participants received a monetary compensation of £10 per h for their participation. The study protocol was approved by the local ethics committee at Queen Mary University of London. Experiments were conducted in accordance with the World Declaration of Helsinki (1964).

#### 2.1.2 Procedure and experimental tasks

##### Free association generation task (FA)

**FIGURE 1:**
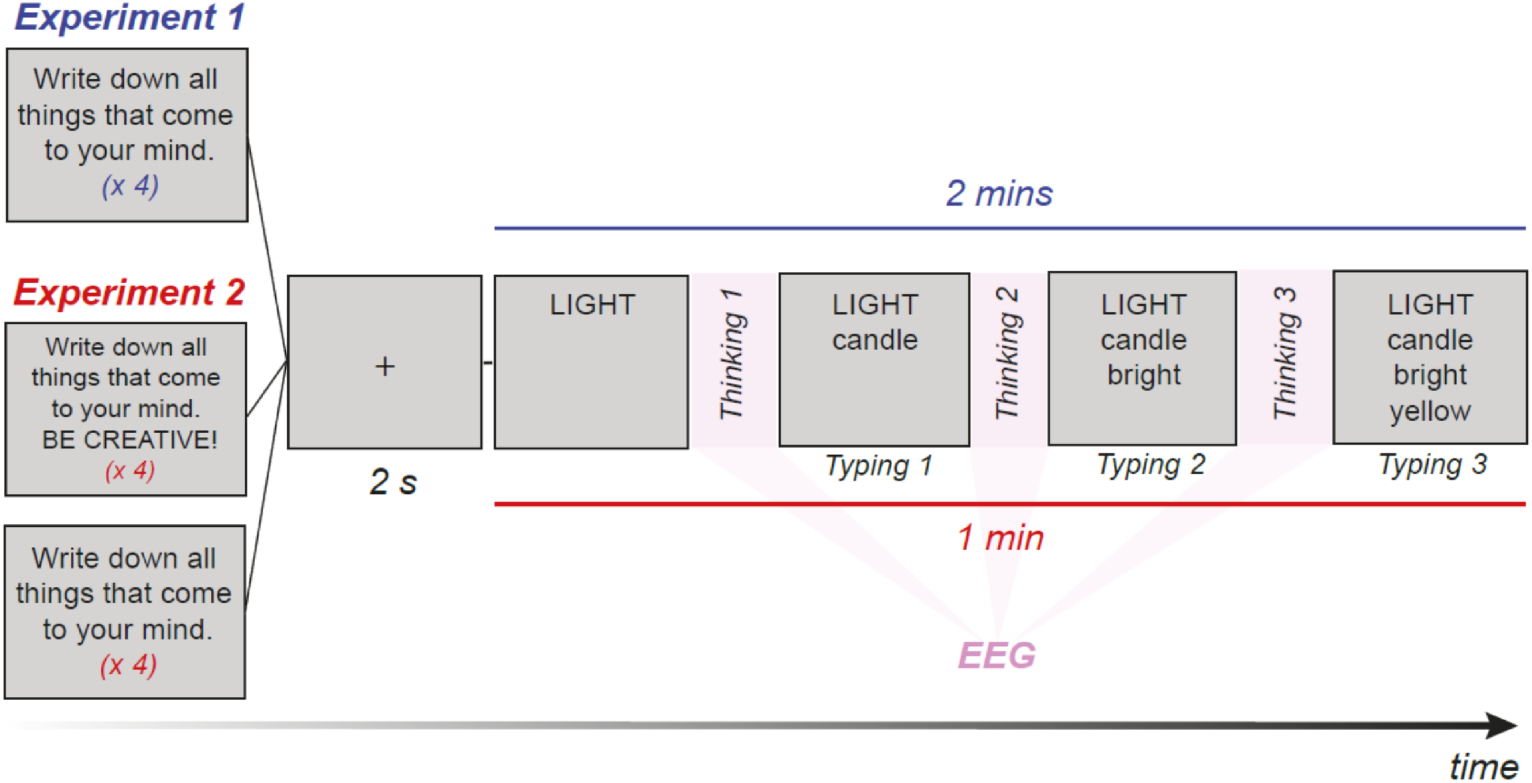
Illustration of the task structure of the free semantic association task. A fixation cross is presented at the centre of the screen for 2 s. Then, the item is presented, and participants type their ideas as they come; therefore, they are thinking and typing interchangeably.

Participants were presented with a word on screen and were given 2 min to type any association that came to mind on a keyboard. After typing, they pressed the enter key to submit their response. They were instructed to type single words only and were encouraged to produce as many associations as possible. The items were the following: king, light, mountain, lion (from Benedek et al., 2012). Before the presentation of each item, a fixation cross was presented at the centre of the screen for 2 s and participants were asked to look at it while resting (Figure 1). The time period between the word cue and the first button press was the “thinking time” used for the EEG analysis (e.g., “thinking 1” in Figure 1), whereas the typing period was not used for the analysis (e.g., “typing 1” in Figure 1). The presentation order of the stimuli was randomised across participants.

#### 2.1.3 EEG recording and preprocessing

The EEG signals were recorded by 18 PiStim electrodes placed according to the extended 10–20 electrode placement system (Jasper, 1958) using a battery‐driven system (StarStim, Neuroelectrics, Spain). The EEG electrodes were: P8, F8, F4, C4, T8, P4, Fp2, Fp1, Fz, Cz, Pz, Oz, P3, F3, F7, C3, T7, and P7. The EEG data were re‐referenced to the algebraic mean of the right and left earlobe electrodes (Essl & Rappelsberger, 1998). Continuous data were high‐pass filtered at .5 Hz and notch‐filtered at 45–55 Hz. Electrodes with poor data quality, as observed by visual inspections, were interpolated from neighbouring electrodes. Artefact rejection was done in a semi‐automatic fashion. Independent component analysis was performed to correct for eye‐blink artefacts. This preprocessing was done using EEGLAB toolbox (Delorme & Makeig, 2004). We then extracted each “thinking time” period before typing onset (response). This time was variable as demonstrated in Figure 1, it depended on the time each response took. We then applied automatic artefact rejection at a threshold of ±80uV. Thinking times shorter than 1 s or with more than 20% of the data tagged as artefact were rejected from the analysis.

#### 2.1.4 Semantic distance analysis

##### Semantic Distance (SD) analysis of the free association generation task (FA)

We assessed the semantic proximity of participants' responses to each given item. Only participants that generated at least 10 associative responses for each of the four items were included in our analysed sample (*N* = 100). We calculated the semantic distances for each response using the “Forward Flow” tool (www.forwardflow.org) (Gray et al., 2019). In this analysis, semantic distances are calculated using latent semantic analysis (LSA) (Deerwester et al., 1990) calculated using a word embedding analysis website (http://wordvec.colorado.edu/). LSA estimates the semantic similarity between two words based on the frequency of their co‐occurrence in a given corpus. Semantic distance is computed as the inverse similarity (1 minus similarity) and ranges from 0 (minimally different) to 1 (maximally different). In our study, we submitted each item separately, so the semantic distance for each response was calculated considering the series of responses within the item (in order). Once we obtained the semantic distances for each response (for each participant and for each condition), we applied a median split on the semantic distance values which enabled us to split the responses into lower and higher semantic distance (for each participant within each condition separately—resulting in a balanced number of responses per participant).

#### 2.1.5 Oscillation detection analysis

Considering that brain oscillations are transient and occur as high‐amplitude short‐bursts and that creativity requires higher‐order cognition which is not precisely time‐locked to the stimulus, we employed an oscillation detection method which enables us to look at these events without assuming stationary signals and without arbitrarily constraining the size of our epochs. Specifically, we adopted a recent improvement of the Better OSCillation Detection (BOSC) framework (Hughes et al., 2012; Whitten et al., 2011), the *f*BOSC (Seymour et al., 2022). This method enables to detangle the arrhythmical background 1/f activity from the rhythmical periods of oscillatory activity. For each participant, we first estimated the background 1/f spectrum based on all the responses (thinking time period—see Figure 1) by applying a Morlet Wavelet transform with 3 cycles from 4 to 40 Hz in steps of 5 Hz. To avoid edge artefacts, we used 0.1 s zero padding. For each frequency, we defined a power threshold based on the modelled background 1/f spectrum, estimated using a spectral parametrisation tool, the fooof (Donoghue et al., 2020). This method enables to model the 1/f spectrum more accurately and to standardise the burst detection across frequency bands. As an optional parameter, we used the knee of the power spectrum. Since oscillations manifest as peaks of power above the periodic signal, we first detected the presence of oscillations by tagging datapoints above 95% of the periodic signal amplitude and with a duration higher than 3 cycles. Within the alpha frequency band, we looked at the most prevalent frequency to identify the spectral peak or the individualised alpha frequency (IAF), separately for each participant. The alpha frequency with the longest duration was considered the individualised alpha peak and used for subsequent analysis. For each response, we calculated (1) the average power of the detected IAF bursts; (2) the proportion of time in which the oscillation was present; (3) the average duration of the IAF bursts. We focused on these three measures since they are relatively independent of the thinking time durations (responses).

#### 2.1.6 Statistical data analysis

Our fBOSC analysis included only participants with at least 10 valid responses in total and at least 5 valid responses in each condition (to avoid excessively imbalanced data and enough data to estimate the 1/f activity). Considering our hypothesis, we extracted the fBOSC measures (listed above) for each response (thinking time) at two key electrodes: T8 (right temporal) and T7 (left temporal). The left temporal was entered as a control, since we expected the effects to be stronger on the right temporal lobe. For each participant, we calculated the average alpha activity (separately for each fBOSC measure) for responses with lower and with higher semantic distance. Our three fBOSC measures (power of detected oscillations, proportion of alpha bursts, average duration of alpha bursts) were entered as dependent variables into a within‐subjects 2 (semantic distance: lower vs. higher) × 2 (hemisphere: left vs. right) MANOVA. The statistical analysis was conducted using Statistical Package for the Social Sciences (IBM SPSS), version 29.0.1 for Windows.

### 2.2 Results

#### 2.2.1 Behavioural results

**FIGURE 2:**
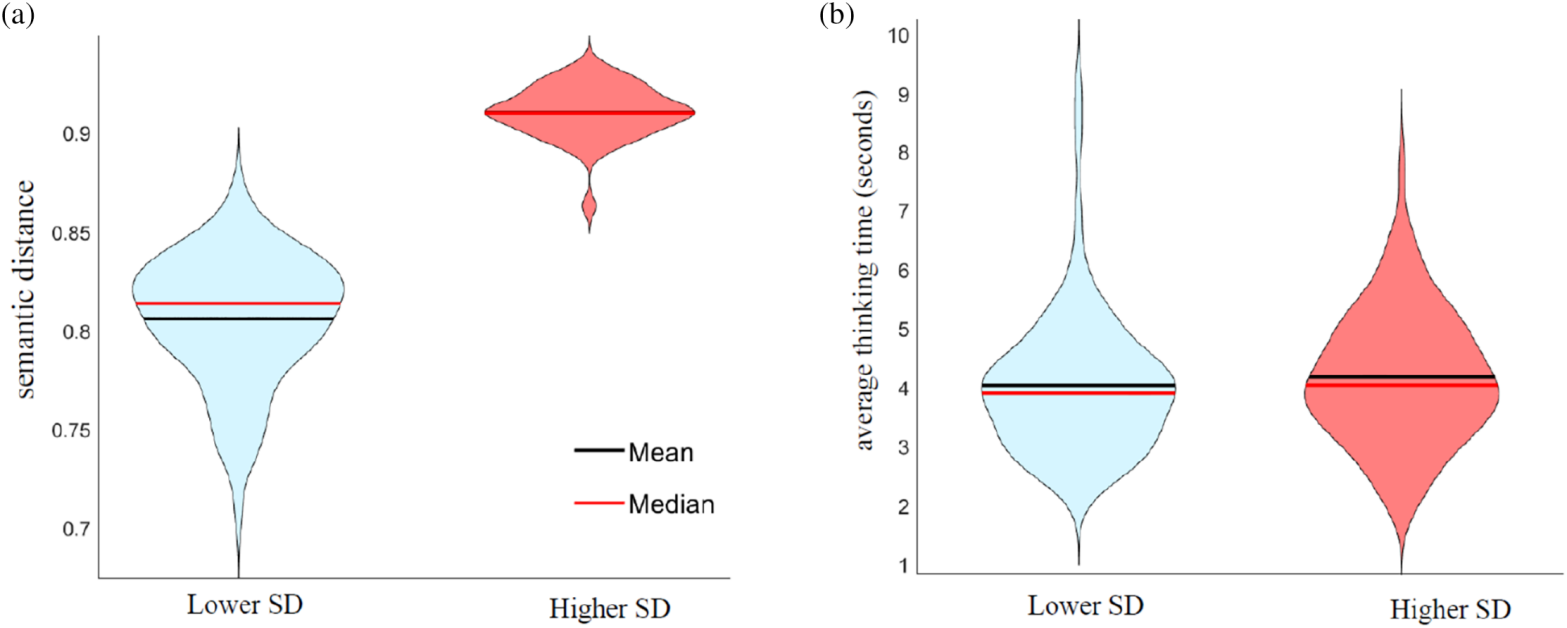
Behavioural findings Experiment 1. Violin plots showing the central tendencies and distributions of the semantic distance (a) and average thinking time (b) for the generation of associations of lower (light blue) and higher semantic distance (light red).

In Figure 2, we present the average and distribution shape of the semantic distances and response times for trials with higher versus lower semantic distances. Since higher and lower semantic distance items were categorised based on the semantic distance itself, we expected a substantial difference in this measure between these two levels, which we observed, *t*(78) = 40.435, *p <* .001. In relation to response times, we observed no significant difference in the response times of items with lower and higher semantic distance, *t*(78) = 1.290, *p = *.201.

#### 2.2.2 Alpha activity during the generation of semantic associations

**FIGURE 3:**
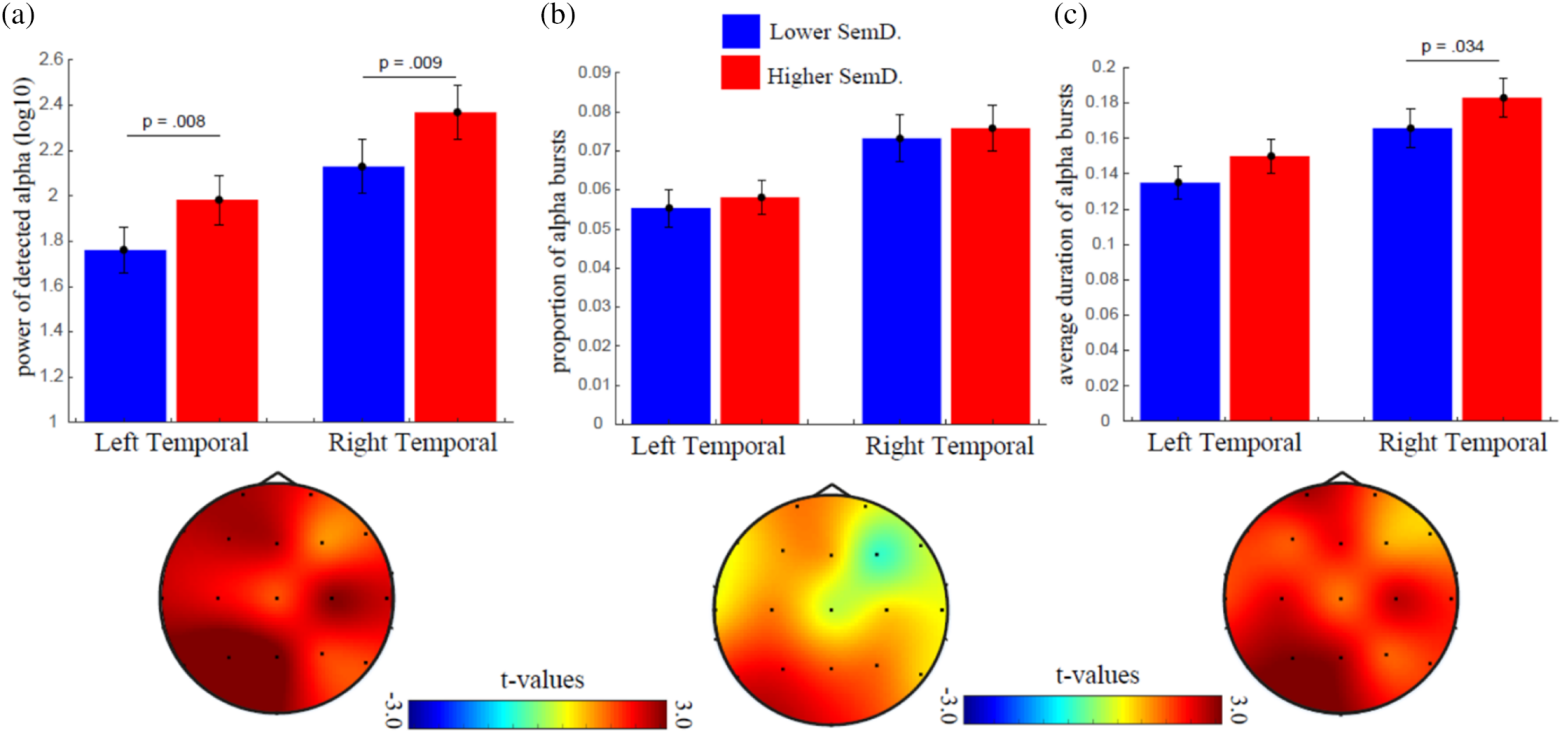
Alpha oscillatory activity during semantic associations. (a) Top: Error bars displaying the power of the detected alpha oscillations on the left (T7) and the right (T8) temporal regions. Bottom: topography of the statistical contrast (*t*‐values) between power of detected alpha oscillations during the generation of associations with higher versus lower semantic distance. Red colours indicate higher power for associations with higher compared to lower semantic distances. (b) Same representations as in A, but for the proportion of alpha episodes. (c). Same representations as in A and B, but for the average duration of the alpha bursts. Error bars represent ±1 SEM.

The distribution of the alpha variables in each condition can be visualised in the supplementary materials (Figure S1). For the EEG, we tested whether alpha oscillations were higher during thinking periods which resulted in semantic associations with lower versus higher semantic distances using a 2 (semantic distance: lower vs. higher) × 2 (hemisphere: left vs. right) MANOVA with 3 dependent variables (power of detected oscillations, proportion of alpha bursts, average duration of alpha bursts), which together represented alpha oscillatory activity. Our analysis revealed that alpha oscillatory activity was significantly higher during the generation of associations with higher semantic distances, *F*(3.76) = 6.888, *p <* .001, partial *η* ^2^ = .214, and that alpha oscillations were most prevalent in the right hemisphere, *F*(3.76) = 7.492, *p <* .001, partial *η* ^2^ = .228, but there was no interaction between the two variables, *F*(3.76) = .024, *p =* .995, partial *η* ^2^ = .001, suggesting that alpha oscillatory activity was more prevalent during the generation of more semantically distant associations independently of the brain hemisphere in the combined three dependent variables, including power of detected oscillations (Figure 3a), proportion of detected burst episodes (Figure 3b), and average burst duration (Figure 3c). Follow‐up univariate tests show that the effect of semantic distance was the strongest for the power of detected alpha oscillations, *F*(1.78) = 11.629, *p =* .001, partial *η* ^2^ = .130, followed by the average burst duration, *F*(1.78) = 6.198, *p =* .015, partial *η* ^2^ = .074, but that it was not significant for the proportion of detected burst episodes, *F*(1.78) = .682, *p =* .411, partial *η* ^2^ = .009. The effect of hemisphere was significant for all three dependent variables, including power of detected alpha oscillations, *F*(1.78) = 21.318, *p <* .001, partial *η* ^2^ = .215, proportion of alpha episodes, *F*(1.78) = 19.812, *p <* .001, partial *η* ^2^ = .203, and average burst duration, *F*(1.78) = 21.795, *p <* .001, partial *η* ^2^ = .218. There was no significant interaction for any of these variables. To improve our interpretation of these results, we plotted the topography of the differences in alpha between lower and higher semantic distances (Figure 3), which shows that alpha was higher during the generation of items with higher semantic distance, but that this effect was widespread rather than localised on the right temporal region.

To control for a potential effect of gender, we conducted the same statistical analysis adding gender as a factor. We conducted a 2 (semantic distance: low vs. high) × 2 (hemisphere: left vs. right) × 2 (gender: female vs. male) mixed‐design MANOVA (3 dependent variables: power of detected oscillations, proportion of alpha bursts, average duration of alpha bursts). Our findings revealed no main effect of gender on any of the dependent variables, including power, *F*(1.77) = 2.386, *p* = .127, partial *η* ^2^ = .030, proportion of alpha bursts, *F*(1.77) = 2.123, *p* = .149, partial *η* ^2^ = .027, and duration, *F*(1.77) = 2.691, *p* = .105, partial *η* ^2^ = .034. There was no significant interaction between gender and any of the factors (semantic distance and hemisphere, *p* > .08 for all the interactions).

### 2.3 Interim discussion

Our findings of Experiment 1 showed that alpha activity was higher during the generation of responses with higher semantic distance; however, this effect was widespread rather than localised on the right temporal region as we initially expected based on previous studies (Camarda et al., 2018; Luft et al., 2018). One key difference between generating free associations and coming up with creative ideas as usually done in creativity tasks is the intention to be creative. In a free association task, spontaneous generative processes do not require the inhibition of closely associated concepts.

We designed Experiment 2 to address the question of whether alpha brain oscillations involved in generating more distant concepts would differ depending on whether the participants are creative intentionally (thus actively inhibiting obvious associations) or spontaneously. We hypothesised that alpha oscillatory activity would be higher during the generation of more distant associations when those are intentionally generated. The inhibition of obvious ideas observed in previous work (Luft et al., 2018) may thus depend on the intention to overcome more obvious associations. Another possibility would be that goal‐directed associations would increase the level of metacognition, promote higher filter/cognitive control (Chrysikou et al., 2014), and engage additional creative processes such as idea evaluation once individuals are asked to come up with creative associations. Since all of these possibilities imply a different level of cognitive control over the creative process, we also investigated directed phase synchronisation in the alpha frequency band. We expected a higher flux from attentional and executive brain regions to right temporal, semantic‐related areas, during the production of free compared to goal‐directed remote associations (i.e., in associations with higher semantic distance).

## 3 EXPERIMENT 2

### 3.1 Methods

#### 3.1.1 Participants

Sixty‐five healthy adults (51 female) aged between 18 and 26 years old (20.53 ± 1.59 years, mean ± SD) took part in the experiment. Six participants were excluded from the analysis due to a low number of responses (less than 10), thus resulting in fifty‐nine participants. All participants received a monetary compensation of £10 per h for their participation. The study protocol was approved by the local ethics committee at Queen Mary University of London. Experiments were conducted in accordance with the World Declaration of Helsinki (1964).

#### 3.1.2 Experimental task

##### Free association task

The free association task in Experiment 2 was identical to Experiment 1 with small modifications: in half of the trials, participants were prompted to generate goal‐directed, creative associations (“Write down all things that come to your mind. BE CREATIVE, avoid the most obvious associations”), whereas in the other half they were prompted to generate free associations (“Write down all things that come to your mind.”). Due to the additional instruction manipulation, the number of cue words increased from 4 to 8 items (clock, lens, soap, stick, lamp, pen, rope, balloon), while the given response time was reduced to 1 min, for each given cue word.

#### 3.1.3 EEG recording

The EEG signals were recorded by a 32‐channel active electrode BioSemi EEG system, following the 10–20 electrode placement system (Jasper, 1958). Pre‐processing of the EEG data was identical to Experiment 1.

#### 3.1.4 Semantic distance analysis

The analysis of the semantic distance was identical to the one conducted in Experiment 1.

#### 3.1.5 Procedures

Participants had their EEG cap placed and signal tested. Once they were ready, they received an explanation about the task in addition to the instructions on the screen. Half of the participants did the task starting with the “be creative” instructions (intentional creative associations) while the other half started with the instructions to just write down what comes to mind first (non‐intentional creativity).

#### 3.1.6 EEG data analysis

Alpha brain oscillations were analysed using the fBOSC method following the exact same procedures and code from Experiment 1.

##### Directed connectivity

Based on our hypothesis that the instruction to be creative could affect the dynamics of the alpha oscillations, which would result in an increase of flux from higher‐order brain areas to the right temporal, we measured directed phase synchronisation in the alpha band using the phase slope index (PSI) (Nolte et al., 2008). The PSI calculates the synchronisation between two signals based on the slope of the phase of their cross‐spectrum. Since the PSI uses the imaginary part of the coherency, it is insensitive to volume conduction. This technique enables the detection non‐instantaneous and directed functional relations between two signals in specified frequency bands. The PSI between channels *i* and *j* was defined as (Nolte et al., 2008):

(1) $${\tilde{\Psi }}_{i,j}=I\left(\sum_{f\in F}C_{i,j}^{*}\left(f\right)C_{i,j}\left(f+\mathrm{\delta f}\right)\right)$$

where

(2) $$\begin{array}{c} C_{i,j}\left(f\right)=\frac{S_{i,j}\left(f\right)}{\sqrt{S_{i,i}\left(f\right)∙S_{j,j}\left(f\right)}} \end{array}$$

is the complex coherency between channels *i* and *j*, S is the cross‐spectral matrix, δf is the frequency resolution of the coherency, and $I\left(\cdot \right)$ denotes getting the imaginary part. F is the set of frequencies over which the slope is summed. The equation is rewritten as follows to see that the definition of ${\tilde{\Psi }}_{\mathrm{ij}}$ corresponds to a meaningful estimate:

(3) $$\begin{array}{c} {\tilde{\Psi }}_{i,j}=\sum_{f\in F}a_{i,j}\left(f\right)\times a_{i,j}\left(f+\mathrm{\delta f}\right)\times \sin\left(\Phi \left(f+\mathrm{\delta f}\right)-\Phi \left(f\right)\right) \end{array}$$

where $a_{i,j}\left(f\right)=\left|C_{i,j}\left(f\right)\right|$ coefficients denote frequency‐dependent weights. For smooth phase spectra, $\sin\left(\Phi \left(f+\mathrm{\delta f}\right)-\Phi \left(f\right)\right)\approx \Phi \left(f+\mathrm{\delta f}\right)-\Phi \left(f\right),$ and hence, Ψ corresponds to a weighted average of the slope. Finally, $\tilde{\Psi }$ is normalised by an estimate of its standard deviation:

(4) $$\begin{array}{c} \Psi =\frac{\tilde{\Psi }}{\mathrm{std}\left(\tilde{\Psi }\right)}\; \end{array}$$

where $\mathrm{std}\left(\tilde{\Psi }\right)$ is the standard deviation of $\tilde{\Psi }$ estimated by the Jackknife method.

#### 3.1.7 Statistical data analysis

##### Brain oscillatory activity

We analysed the same three main measures of alpha oscillatory activity: average power of detected alpha oscillations, proportion of alpha oscillatory episodes or bursts against thinking time duration, and average burst duration (the arithmetic mean duration of each alpha burst). We entered these three dependent variables into a MANOVA with the following factors: 2 (semantic distance: lower vs. higher) × 2 (creative intention: free‐ vs. goal‐directed) × 2 (hemisphere: right vs. left). We excluded participants with less than five usable trials in any of the conditions (*n* = 5) and extreme outliers (*n* = 4), resulting in a total sample of 50 participants. Extreme outliers were defined as being above 3 standard deviations of the group/condition mean.

##### Directed connectivity

To investigate how the goal‐directed intention to “be creative” affected alpha oscillatory activity dynamics, we calculated directed connectivity within the alpha band using the Phase Slope Index (PSI). Since there were no previous studies on alpha‐directed connectivity during idea generation, we used a data‐driven approach, a non‐parametric cluster permutation on the PSI connectivity matrix. The non‐parametric cluster permutation test (Maris & Oostenveld, 2007) was used to compare the synchronisation of alpha oscillatory activity during the generation of associations with higher versus lower semantic distances, but especially focusing on the differences in connectivity between free‐ versus goal‐directed during the generation of associations with higher semantic distance. We followed the same procedures used in our previous connectivity work (Luft et al., 2022), which combines traditional nonparametric cluster permutation with graph theory to estimate the clusters. In the non‐parametric cluster permutation approach, we eliminate potential biases introduced by multiple comparisons and distribution assumptions of parametric tests, by contrasting our connectivity matrices against a random distribution obtained based on label randomisations combined with a network‐based clustering criterion for the *t*‐statistic extraction (Zalesky et al., 2010). We control for family‐wise error rate without sacrificing statistical power by considering the topological characteristics of the network. The assumption is that a biologically meaningful effect on the network is unlikely to be present on single or disconnected edges. Biologically relevant clusters are expected to display connected components (linked to each other).

To conduct this analysis, we first calculated the statistical contrast for each PSI edge (e.g. all‐to‐all free‐ vs. goal‐directed comparisons), discarding non‐significant *t*‐values (*p* > .05). The surviving edges are then clustered in the so‐called “strong connected components” (SCCs; partition into subgraphs with the property of having at least one path between all pairs of nodes) depending on whether they reflect identical effects (separate clusters for positive and negative edges). Importantly, we considered whether the cluster was higher for one condition or the other by looking at the absolute PSI values. For example, if the *t*‐values between connections of a cluster are positive for free‐ versus goal‐directed associations, the positive value alone cannot inform us about the nature of the connections underlying the statistical effect. To deal with this problem, the cluster membership was assigned depending on the absolute value of the PSI. If directed connectivity was higher in the intentional condition, that edge would be assigned to the intentional cluster and vice versa. This enabled us to distinguish between potential connectivity clusters. Subsequently, different distribution curves of the condition differences were estimated using 5000 permutations per frequency band by randomly shuffling the condition labels (e.g. intentional and non‐intentional), without modifying each participant's data. In each iteration, the sum of *t*‐scores within each cluster was calculated and kept the maximum (absolute value) cluster score (called cluster *t*‐statistic). The distribution of the cluster *t*‐statistics (derived from all iterations) was then used to calculate the *t*‐critical values at the significance level of .05 (two‐tailed). The *t*‐scores of data clusters formed by the actual labels were then compared to this distribution. Cluster's *t*‐scores which exceeded the *t*‐critical values were deemed significant. The corresponding *p‐*values were reported alongside the *t*‐scores.

### 3.2 Results

#### 3.2.1 Behavioural results

**FIGURE 4:**
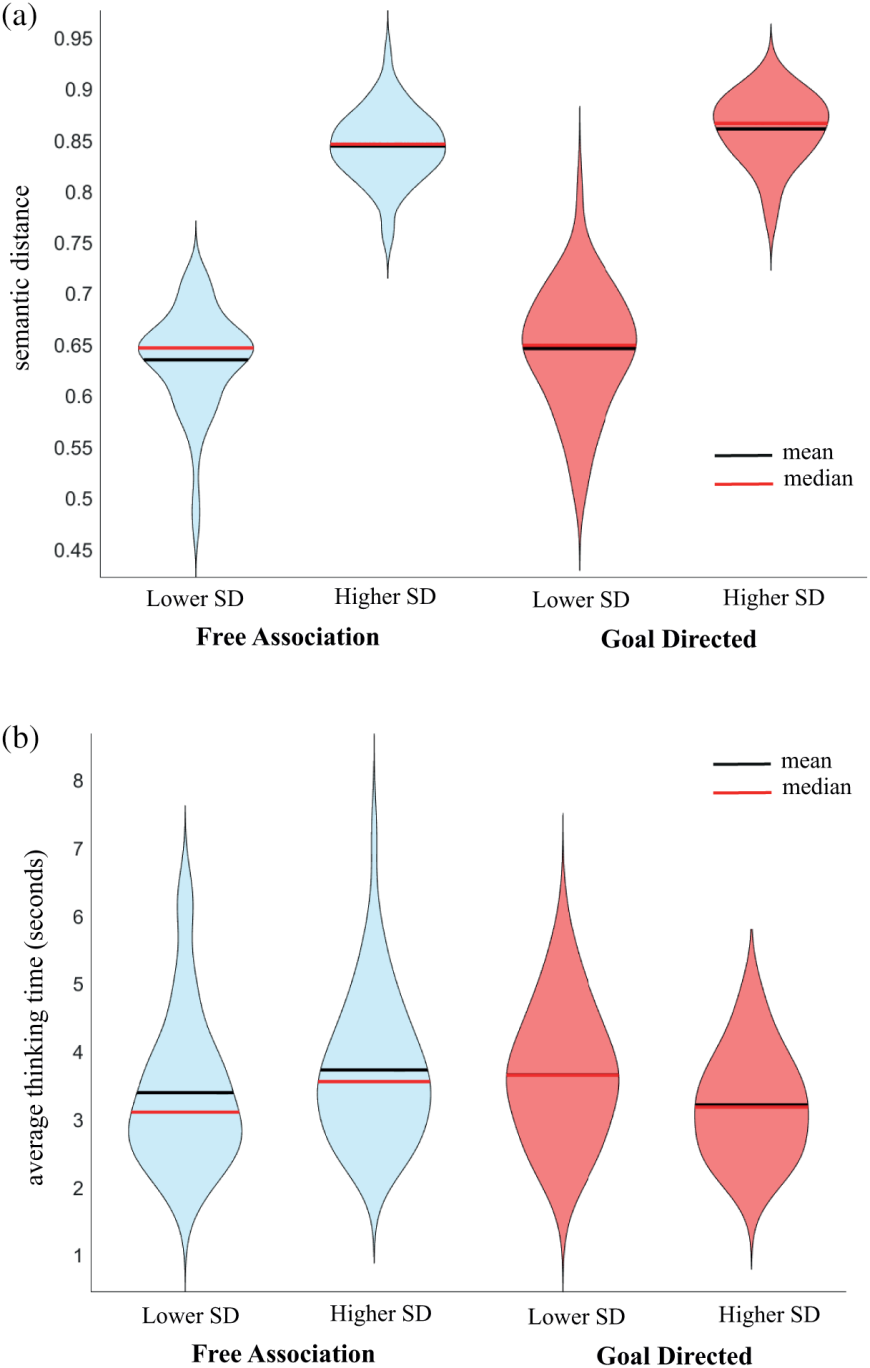
Behavioural results Experiment 2. Violin plots showing the central tendencies and distributions of the semantic distance (a) and average thinking time (b) for the generation of the free (blue) and goal‐directed (red) associations.

In Figure 4, we present the average and distribution shape of the semantic distances and response times for trials with higher versus lower semantic distances for the free association and goal‐directed conditions. We conducted two 2 (semantic distance: higher and lower) × 2 (creative intention: free‐ vs. goal‐directed) repeated measures ANOVAs, one using the semantic distance and the other using response times as dependent variables. Since higher and lower semantic distance items were categorised based on the semantic distance itself, we expected a main effect of semantic distance, which we observed, *F*(1.49) = 2315, *p <* .001, partial *η* ^2^ = .979. We also observed a marginally significant effect of intention, *F*(1.49) = 4.326, *p =* .043, partial *η* ^2^ = .081, since goal‐directed associations were more remote. There was no interaction between semantic distance and creative intention, *F*(1.49) = .474, *p =* .494, partial *η* ^2^ = .010 (Figure 4a). In relation to response times, we observed no significant main effects of semantic distance, *F*(1.49) = .127, *p =* .723, partial *η* ^2^ = .003, nor of creative intention, *F*(1.49) = 1.342, *p =* .252, partial *η* ^2^ = .027, suggesting that the participants took a similar time to come up with goal‐directed and free associations. However, there was a significant interaction between semantic distance and creative intention. *F*(1.49) = 10.434, *p =* .002, partial *η* ^2^ = .176, as the response times were substantially lower during goal‐directed associations with higher semantic distance as shown in Figure 4b.

#### 3.2.2 Alpha activity during the generation of semantic associations

We tested the differences in alpha oscillations in the temporal areas during the generation of ideas with lower versus higher semantic distance when in free‐ versus goal‐directed association tasks. We hypothesised that right temporal alpha would be increased during the generation of associations with higher semantic distance, but only when participants were attempting to be creative in a goal‐directed fashion.

**FIGURE 5:**
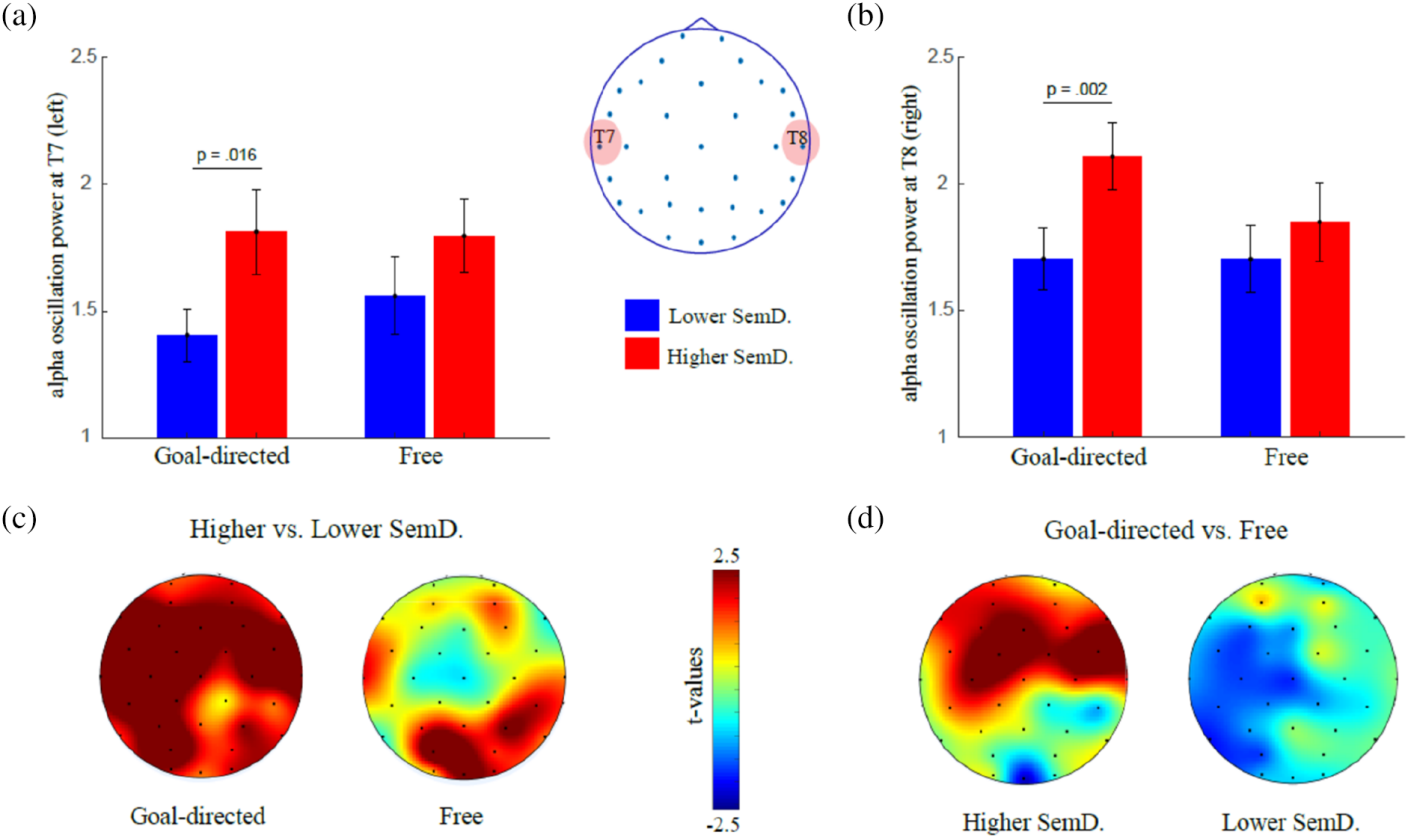
Power of detected alpha oscillations during the generation of semantic associations. A/B. Power of detected alpha oscillations during the generation of associations with lower (below median, blue) and higher (above median, red) semantic distance during free‐ and goal‐directed conditions. (a). In the left temporal area (electrode T7); (b). in the right temporal area (electrode T8). (c). Topography of the differences in power of detected alpha oscillations during the generation of higher versus lower semantic distance. Red colours indicate larger alpha power during the generation of items with higher compared to lower semantic distance in the goal‐directed (left) and free (right) conditions. (d). Topography of the differences in power of detected alpha oscillations during the generation of word associations in the free‐ versus goal‐directed conditions for associations with lower (left) and higher (right) semantic distance. Red colours show larger alpha during goal‐directed compared to free association generation conditions. The colour bar applies to both C and D topomaps and represents the statistical contrast between the conditions (*t*‐values). Error bars represent ±1 S.E.M.

We conducted a 2 (semantic distance: lower vs. higher) × 2 (creative intention: free‐ vs. goal‐directed) × 2 (hemisphere: right vs. left) within‐subjects MANOVA using alpha power, proportion of alpha oscillatory activity and average alpha burst duration as the dependent variables. We observed a main effect of semantic distance, *F*(3.47) = 6.410, *p <* .001, partial *η* ^2^ = .290, since alpha was higher for generating word associations with higher semantic distance in both hemispheres and in both conditions (free‐ and goal‐directed). There was no main effect of creative intention, *F*(3.47) = 1.409, *p =* .252, partial η^2^ = .083, nor hemisphere, *F*(3.47) = 2.151, *p =* .106, partial *η* ^2^ = .121. There was no significant interaction (*p >* .05). The univariate ANOVA showed that the effect of semantic distance was significant in all dependent variables, including power of detected alpha oscillations, *F*(1.49) = 17.505, *p <* .001, partial *η* ^2^ = .263, mean duration of the alpha burst, *F*(1.49) = 10.197, *p =* .002, partial *η* ^2^ = .172, and proportion of alpha oscillatory activity, *F*(1.49) = 6.237, *p =* .016, partial *η* ^2^ = .113. The differences in alpha oscillation power can be observed in Figure 5a (left temporal) and Figure 5b (right temporal).

Since our analysis was focused on the temporal region, we also present the full topography of the differences in alpha oscillations for items with lower and higher semantic distance and for the ones generated in the free‐ versus goal‐directed conditions. The topographies show (Figure 5c) that items with higher semantic distance were associated with higher widespread alpha power, especially in the goal‐directed condition. Importantly, the generation of higher semantically distant associations was associated with more localised increase in alpha power in the anterior‐temporal region, extending all the way to right fronto‐lateral (Figure 5d). This increase was more salient during the generation of more distant associations. Similar findings were observed for the other two dependent variables and are presented in the Supplementary Materials (Figures S3 and S4). To control for a potential effect of the order of the conditions (goal‐directed vs. free‐association first), we conducted the same MANOVA adding order as a factor. The results show (Supplementary Materials S5) no effect of order or interaction with creative intention and semantic distance.

#### 3.2.3 Directed connectivity during generating goal‐directed creative semantic associations

We conducted two analyses. First, we compared the PSI values during goal‐directed generation of more distant associations with less semantic distant ones (i.e. higher vs. lower semantic distance). We expected that synchronisation in the alpha frequency band will be directed towards the right temporal hemisphere and higher during the generation of more distant semantic associations. Second, we compared the PSI values between the free‐ versus goal‐directed conditions in the items with higher semantic distance. Since PSI values can be positive or negative, we calculated the clusters separately considering whether the connections were positive or negative and whether they were higher during free‐ or goal‐directed associations. For example, if we found a positive cluster, meaning higher PSI during the goal‐directed condition was higher than during the free‐association condition, we checked whether the difference was due to higher negative PSI values in the free‐association condition or whether it was because of higher PSI values in the goal‐directed condition.

For the first analysis, we observed a marginally significant cluster in the contrast of directed alpha synchronisation during the generation of associations with higher > lower semantic distance (goal‐directed associations only: *t*‐statistics = 72.5443, *t*‐critical = 80.5016, *p =* .057). There was also no significant cluster in the opposite direction (lower semantic distance > higher semantic distance: *t*‐statistics = 16.8892, *t*‐critical = 80.5016, *p =* .23).

**FIGURE 6:**
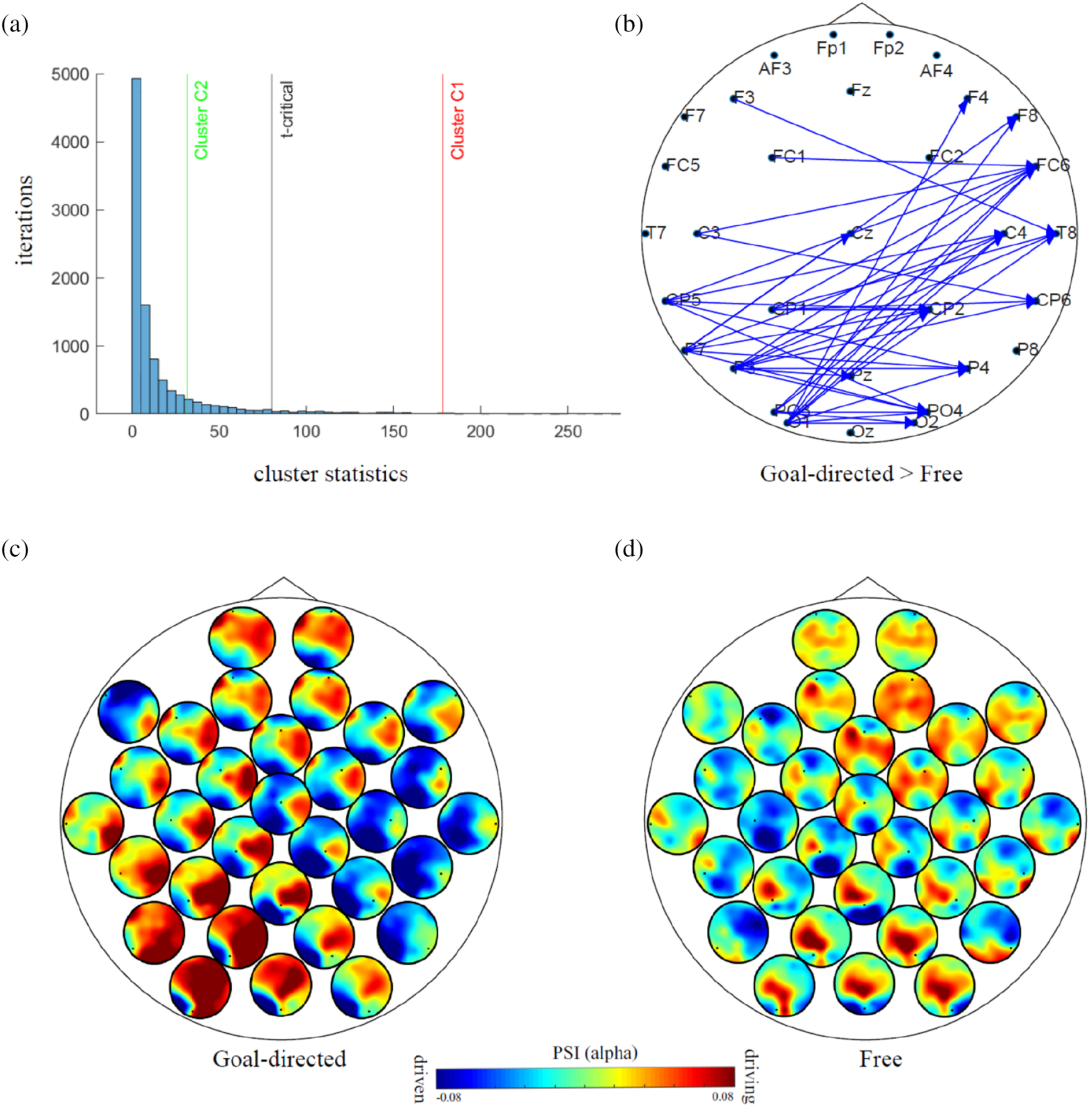
Directed synchronisation during free‐ and goal‐directed higher distance semantic associations. (a). Non‐parametric cluster permutation results displayed as a histogram displaying the distribution of the cluster statistics (sum of cluster *t*‐values) for all 5000 randomly shuffled iterations and the *t*‐critical (black line at alpha level = .05) compared to the real cluster statistics for goal‐directed > free (red), free > goal‐directed (green) association contrasts. (b). Significant directional links of the goal‐directed cluster (the display only includes links with *p* < .025). The arrows show the direction of the connections. (c). Heads‐in‐head plot showing the topography of the directed phase synchronisation (PSI) during goal‐directed (left) and free (right) associations with higher semantic distance. The dots inside each circle (heads) represent the channel, whereas the colours inside the head represent the topography of the connections with this channel (dot). Blue colours indicate that this channel is being driven by these areas while red colours indicate that this channel is driving the areas in red. For example, in the goal‐directed heads‐in‐head plot, the right temporal electrode (T8) is being driven by most areas in the left hemisphere.

For our second analysis, we observed a significant cluster for goal‐directed > free associations (*t*‐statistics = 178.38, *t*‐critical = 80.1797, *p =* .01), but no significant cluster in the opposite direction (free > goal‐directed associations: *t*‐statistics = 31.39, *p* = .15). The statistical significance against the shuffled distribution can be observed in Figure 6a. The significant cluster shows a robust flux of synchronisation from the left to the right hemisphere (Figure 6b) during the goal‐directed compared to the free remote semantic associations. To inspect whether this significant cluster could have emerged from the contrast rather than an increased in flow from left to right, we plotted the entire directed synchronisation topography for each condition separately (Figure 6c,d). It is evident in the heads‐in‐head plots that there was a higher increase in PSI from left to right in the goal‐directed associations, whereas in the free associations there was no clear synchronisation pattern.

We conducted the same cluster analyses on the free associations (higher vs. lower semantic distance), but there was no significant cluster in either direction (*p* > .05). Similarly, we conducted the same non‐parametric cluster analysis comparing goal‐directed versus free associations generation of lower semantic distance items and observed no significant cluster in either direction (*p* > .05). These findings suggest that directed synchronisation in the alpha band is stronger during successfully goal‐directed inhibition of closer semantic associations.

Finally, we conducted the same cluster permutation analysis using the data of Experiment 1 (free association only), contrasting higher versus lower semantic distance alpha synchronisation. In line with Experiment 2, we found no statistically significant directed alpha synchronisation cluster between lower and higher semantic distance associations, including non‐significant higher > lower semantic distance (*t*‐statistics = 3.1894, *t*‐critical = 19.8081, *p* = .34) and non‐significant lower > higher semantic distance (*t*‐statistics = 7.9143, *t*‐critical = 19.8081, *p* = .17).

## 4 DISCUSSION

To the best of our knowledge, this is the first study investigating the role of alpha oscillations in free and goal‐directed (by instruction) semantic association tasks. We observed a widespread increase in alpha oscillatory activity during the generation of more distant semantic associations. Interestingly, we observed that this increase in alpha oscillatory activity was more robust when people were told to be creative. Furthermore, we observed that alpha synchronised from the left to the right hemisphere during the goal‐directed generation of more distant semantic associations, suggesting that the goal‐directedness of the instruction to be creative might change the flux of information in the brain during remote idea generation.

In a recent review, Beaty and Kenett (2023) highlight the role of associative thinking as a core mechanism of creativity, both general and domain‐specific. Importantly, the authors distinguish between free‐, spontaneous or unintentional, versus goal‐directed, intentional associations, and how both types of associations are relevant in creativity. They emphasise that such associative processes operate over semantic memory and are considered to facilitate the ability to connect remote ideas, which some consider to be the core of the creative process (Abraham & Bubic, 2015; Benedek et al., 2023; Mednick, 1962). Therefore, elucidating the neural mechanisms related to generating close and remote associations in free‐ and goal‐directed manners is critical to further elucidate associative thinking in general, and specifically the creative thinking process. However, it is important to acknowledge that the creative thinking process includes many additional processes, such as attention and metacognitive processes (Benedek & Fink, 2019; Green et al., 2023; Lebuda & Benedek, 2023).

Our work offers four main contributions to the wider literature in cognitive neuroscience and in creativity: (1) there is a vast literature on brain oscillations and creativity (e.g., Agnoli et al., 2020; Benedek et al., 2011; Camarda et al., 2018; Eymann et al., 2022; Fink & Neubauer, 2006; Luft et al., 2018; Mastria et al., 2021; Perchtold‐Stefan et al., 2020; Sandkühler & Bhattacharya, 2008; Yu et al., 2023); however, this is the first study to investigate brain oscillatory activity during the generation of free and goal‐directed semantic associations. This is important since such tasks are widely used in different fields, especially in creativity research, but we know very little about the underlying processes (Beaty & Kenett, 2023; Benedek et al., 2023); (2) this is also the first study to compare brain oscillations during goal‐directed versus free semantic associations. This is a key contribution of our study since often semantic distance in free association tasks is commonly used as a measure of creativity due to significant but weak to moderate correlations with traditional creativity measures (Beaty & Johnson, 2021; Dumas et al., 2021; Organisciak et al., 2023). Here we show that free and goal‐directed association tasks might rely on different neural processes; (3) this is also the first study to use an oscillation detection method to investigate brain oscillatory activity during association generation, as previous studies either used time‐frequency representations or frequency domain transformations (e.g., FFT, wavelets). Beyond the superiority of such method to separate rhythmic brain oscillatory activity from 1/f background activity, this method also enabled us to break free from the assumptions: (a) that the idea generation process is stationary (i.e. remains the same during the entire trial duration); (b) that the average power spectrum represents the dynamic of the thinking processes; (c) that these processes occur at the same time after the presentation of the stimulus (i.e., that they are time‐locked to events such as the presentation of cue words). For long, we have been aware of the limitations of the more traditional power spectral methods (e.g., FFT, wavelets) when analysing higher‐order creativity processes, but have not applied oscillation detection methods which can help overcome these limitations; (4) our two experiments demonstrate that alpha oscillatory activity is consistently higher during more remote associations, but that the topography of the effects is widespread rather than localised, which helps to explain the lack of consistency of previous studies on creativity in terms of brain regions. This finding is key as it shows that perhaps interpreting complex creativity processes (e.g., idea generation) as a result of specific brain regions or even hemispheres might be inaccurate.

In line with the literature on alpha oscillatory activity during creativity tasks (Agnoli et al., 2020; Benedek et al., 2011; Camarda et al., 2018; Fink & Benedek, 2014; Fink & Neubauer, 2006; Luft et al., 2018; Mastria et al., 2021; Perchtold‐Stefan et al., 2023; Stevens Jr & Zabelina, 2019), we observed that alpha was consistently higher during more distant semantic associations (in both studies, there was a main effect of semantic distance). However, we did not find that this effect was specific to any brain region, especially not to the right temporal area as observed in previous creativity studies (Camarda et al., 2018; Luft et al., 2018). In Experiment 2, we tested the hypothesis that such topographically specific effect would be clear only when the intention to be creative was present (as it is the case in most creativity tasks), especially since this intention would require to actively inhibit more obvious semantic links. That hypothesis was not confirmed since the effect was very strong on most frontal and temporal areas (bilaterally), including the right antero‐temporal areas. This could mean that alpha oscillations in the right temporal area might reflect the integration of different concepts as required in more complex creativity tasks, such as the alternative uses task (Acar & Runco, 2019), beyond the inhibition of obvious ideas (as previously suggested by Luft et al., 2018). Indeed, the anterior temporal lobe (left hemispheric but also right hemispheric to a lesser extent) has been widely associated with semantic composition during combinatorial operations (Del Prato & Pylkkänen, 2014; Segaert et al., 2018; Westerlund & Pylkkänen, 2014).

Alternatively, lower alpha oscillatory activity during the generation of less remote ideas could be related to the specific conceptual activation of an obvious association. An EEG study found that silent gaps before predictable final words of sentences were associated with alpha/beta decreases, supporting the pre‐activation of linguistic information (Gastaldon et al., 2020). Furthermore, in a picture naming task embedded in constraining versus non‐constraining sentence contexts, lower alpha/beta power has been observed for the constraining condition, interpreted as conceptual‐lexical retrieval from memory (Cao et al., 2022; Piai et al., 2014). Crucially, this effect disappeared in the case of broad conceptual activation, instead of a narrow specific conceptual retrieval (Hustá et al., 2021). These power decreases have also been found prior to the presentation of words in strongly versus weakly constraining sentences without response verbalisation, suggesting that these effects are not linked to naming or articulatory operations (Rommers et al., 2017). Overall, the aforementioned findings could suggest that our observed increased alpha activity during generation of semantically distant associations might reflect a broader conceptual activation, which could be required in order to reach more remote associations.

Notwithstanding the widespread differences in alpha oscillations between our experimental conditions, both experiments indicate that alpha is substantially higher in the left parietal regions during the generation of more distant associations. Both experiments showed that alpha was also significantly higher in the right temporal, especially antero‐temporal, regions. Considering that our study utilised EEG, which has very limited spatial resolution, we cannot discuss our findings in terms of anatomical regions (see Ovando‐Tellez et al., 2023). However, we can suggest that the effect is widespread and likely to involve a distributed network of regions which includes frontal, temporal, and posterior regions, as it is the case in semantic cognition (Jefferies, 2013; Noonan et al., 2013; Ralph et al., 2017), especially semantic control (Jackson, 2021; Thompson et al., 2022).

Association tasks rely on semantic cognition more broadly, including semantic representation and semantic control processes (Fradkin & Eldar, 2023; Lerner et al., 2012, 2014). Semantic representation is defined as the extraction and storage of the structure of the environment whereas semantic control is the ability to selectively access and manipulate meaningful information according to context (Jackson, 2021; Ralph et al., 2017). Independent of the instruction, both experiments required participants to access their semantic representations. On the other hand, semantic control was increased by our instruction to be creative, since participants had the additional task to filter out or inhibit close associations, which come to mind first. Thus, our experiments provide electrophysiological evidence for the interaction between semantic representations and the processes that operate on them (Beaty et al., 2023; Beaty & Kenett, 2023; Marko & Riečanský, 2021; Michalko et al., 2023).

Interestingly, we observed that alpha oscillatory activity was stronger when participants generated associative responses with higher semantic distances. However, alpha oscillations were higher when participants generated these associative responses intentionally, in a goal‐directed manner. Task‐related alpha oscillations are often interpreted as representing a process of active inhibition of task‐irrelevant regions (Klimesch, 2012; Klimesch et al., 2007). However, when observed in task‐relevant regions, alpha oscillations are thought to represent a process of selective access to knowledge systems by inhibition (Klimesch, 2012). The need for selective access to knowledge systems is higher when there is the intention to be creative. When generating semantic associations under this instruction, participants exhibited substantially higher alpha oscillations, especially on the frontal (bilateral) and right antero‐temporal regions. We suggest that this effect relates to the increased top‐down control to access the semantic representations which were more remote and require people to suppress closely related associations. Thus, our findings relate to the matched filtering hypothesis of cognitive control, which argues that cognitive control is utilised as a filter, based on task demands (Chrysikou et al., 2014).

We also observed that when generating more semantically distant associations under the instruction to be creative, participants showed an increase in long‐range synchronisation from left–left posterior regions but also frontal—to the right temporal area in the alpha band. This difference was not present in the contrast between associations with higher compared to lower semantic distance, which suggests that this directed synchronisation effect may be linked to the need for increased semantic control. Considering that the right temporal area is fundamental for representing semantic knowledge (Jung‐Beeman, 2005; Lambon Ralph et al., 2010; St George et al., 1999; Tranel et al., 1997) and that semantic control areas are mostly on the left hemisphere (Binder et al., 2009; Jackson, 2021), we suggest that the instruction to be creative enables more selective access to knowledge systems by increasing the communications from semantic control areas on the left to semantic representation areas on the right hemisphere. It is reassuring that the communication between these areas also happens in alpha which could mean that it is used to improve the communication between relatively distant areas and enable the binding of the information across them.

Finally, our findings directly relate to a recent study (Ovando‐Tellez et al., 2022). examining switching and clustering process in memory recall via free association, devising a task using polysemous words—that is, words with multiple meanings (e.g., bark; dog‐bark, tree bark)—which enabled clear distinctions for switching and clustering (e.g., naming dog‐related words and not tree is clustering). In this study, they observed that switching correlated with convergent thinking (e.g., connecting remote concepts), whereas clustering correlated with divergent thinking (e.g., generating different ideas). Moreover, switching is related to both executive functions and semantic memory network structure, whereas clustering is related to the ability to retrieve many items from a semantic category (i.e., fluency). These findings indicate that switching during free association reflects an interplay between controlled processes and semantic memory network structure (i.e., “exploration” of semantic space), and clustering reflects controlled processes relevant for persistent/exhaustive memory search (i.e., “exploitation” of a semantic category). This relation between switching and semantic memory network structure provides additional evidence that associative thinking reflects a search process operating on a semantic memory network structure (Beaty & Kenett, 2023). Critically, the findings of Ovando‐Tellez et al. (2022) highlight how cognitive search is based on goal‐directed attention mechanisms, which corresponds to our current findings.

Recent application of computational tools have advanced the research of semantic representations in relation to creativity (Kenett, 2024; Kenett & Faust, 2019), as well as the processes operating over these representations (Beaty & Kenett, 2023). For example, He et al. (2020) triangulated the relations between associative thinking, semantic memory structure, and creative thinking, finding that associative thinking mediated the relationship between semantic memory network efficiency and creative ability. In other words, higher creative people could search through semantic space more fluently, and make more distant semantic associations, because they possessed a more richly connected semantic memory network (Beaty & Kenett, 2023). Considering that in our study we demonstrated that constraining the associations to the more creative/distant ones was associated with a different direction of phase synchronisation, future studies could investigate whether more creative individuals have a stronger flux from cognitive control regions to the right temporal or semantic regions, which could indicate a stronger ability to selectively access their knowledge systems. This would also result in higher alpha oscillatory activity under the generation of ideas which are intentionally creative and while suppressing obvious or closely related concepts.

One important factor to consider when comparing oscillatory activity between free and goal‐directed associations is that the semantic distance of the associations generated under the instruction to be creative will be inevitably higher, creating a potential confound in the analysis. In our study, the main effect of creative intention was on the directed connectivity, and although we did not find an effect for semantic distance on connectivity, it is possible that it contributed to the cluster we observed. Another limitation of our study is the lack of spatial resolution due to the use of EEG methods. We constrained our analysis to the electrode space, which hinders our ability to interpret our findings in terms of brain regions. Furthermore, we had a limited number of trials, which resulted in a large number of excluded participants on the first experiment.

We conclude that alpha oscillatory activity is higher during the generation of more remote semantic associations in association tasks. This effect is not topographically localised and seems to vary largely between participants and experimental groups. Importantly, instructing participants to be creative in a goal‐directed association task was associated with a substantial change in the connectivity patterns, from the left hemisphere to right temporal regions. This finding suggests that the instructions to be creative might have a profound effect on the neural mechanisms behind the generation of creative associations.

## FUNDING INFORMATION

The corresponding author would like to acknowledge support from the BIAL Foundation (No. 138/18).

## CONFLICT OF INTEREST STATEMENT

There is no conflict of interest.

## Supporting information


**Data S1.** Supporting information.

## Data Availability

The data underpinning this publication are available at https://osf.io/gra42 under a CCBY licence https://doi.org/10.17605/OSF.IO/GRA42.
