# Supplementary material for: The role of alpha oscillations in free‐ and goal‐directed semantic associations
Source: Hum Brain Mapp. 2024 Jul 5;45(10):e26770. doi: 10.1002/hbm.26770 (PMC11226545; doi:10.1002/hbm.26770)
Supplement: Supplementary file 1 — Data S1. Supporting information. [file HBM-45-e26770-s001.pdf]

Supplementary Information for

The role of alpha oscillations in free- and goal-directed semantic associations

Ioanna Zioga[1], Yoed N. Kenett[2], Anastasios Giannopoulos[3], Caroline Di Bernardi Luft[4]

Affiliations:

[1] Donders Institute for Brain, Cognition and Behaviour, Radboud University, Nijmegen, The Netherlands

[2] Faculty of Data and Decision Sciences, Technion - Israel Institute of Technology, Haifa Israel

[3] School of Electrical and Computer Engineering, National Technical University of Athens (NTUA) Athens, Greece

[4] Division of Psychology, CHMLS – Life Sciences, Brunel University London, London, UK

Corresponding author:

Caroline Di Bernardi Luft

E-mail address: caroline.luft@brunel.ac.uk

ORCID ID: 0000-0002-3293-3898

This file includes:

S1: Alpha Oscillations data distribution Study 1

S2: Alpha Oscillations data distribution Study 2

S3: Proportion of Alpha Oscillatory Episodes

S4: Average alpha burst duration

S5: Controlling for order of conditions in Study 2

S1: Alpha oscillations data distribution: Study 1

We tested the normality distribution of the dependent variables using Kolmogorov-Smirnov test of normality. Power was found to be normally distributed in both hemispheres and conditions ($p > .05$), whereas proportion of alpha bursts and duration did not follow a normal distribution ($p < .05$). Since not all the variables were normally distributed, we tested the main effects (contrasts between Lower and Higher Semantic distances) presented on Figure 2, using the non-parametric Wilcoxon test. Similarly to what we found using parametric contrasts (Fig.2), we observed a significant difference between high and low semantic distance in alpha power on the right ($Z = 2.644$, $p = .008$) and on the left temporal area ($Z = 2.501$, $p = .012$). The duration of alpha bursts was higher during the generation of associations with higher semantic distance on the left temporal ($Z = 2.145$, $p = .032$), but not significantly on the right temporal ($Z = 1.951$, $p = .051$).The distribution of each of these variables can be visualised in the violin plots presented in Fig.S1.

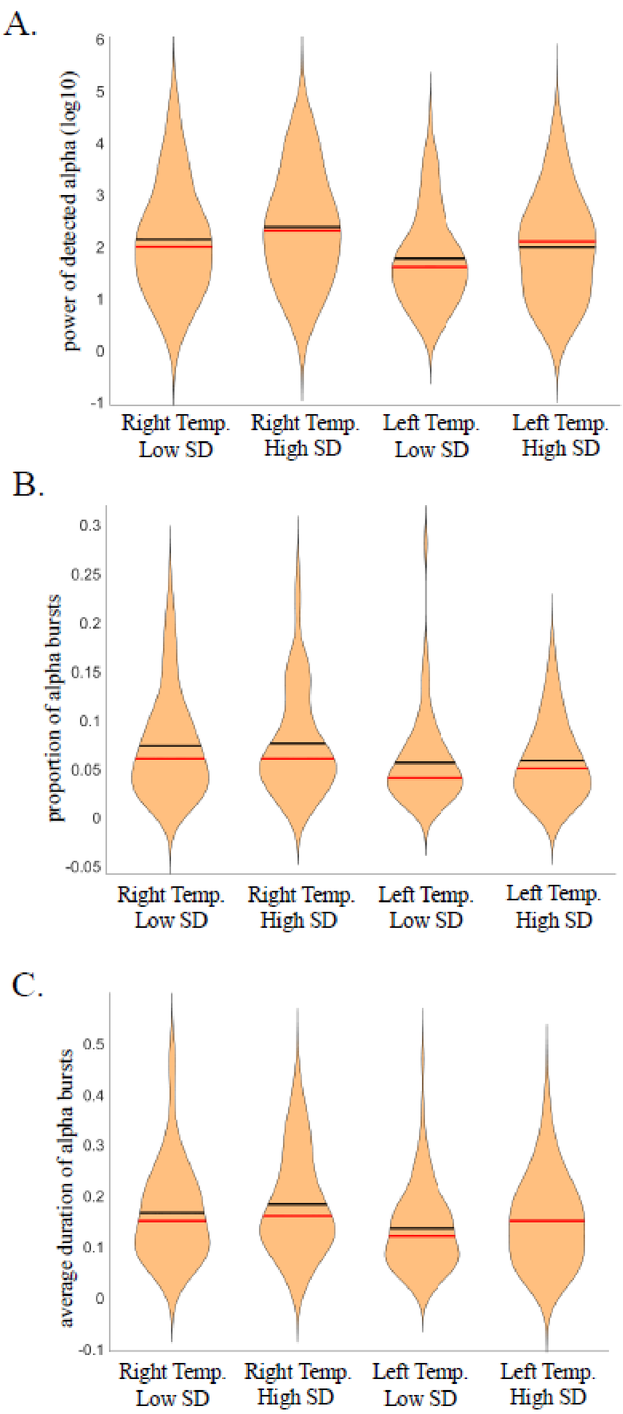

Fig.S1. Violin plots demonstrating the distribution of the three dependent variables in each condition, including: A. Power of detected alpha oscillations (log10 transformed); B. Proportion of alpha bursts; C. Average duration of alpha bursts. Mean is represented with a black line, and median with a red line.

S2: Alpha oscillations data distribution: Study 2

We tested the normality distribution of the dependent variables using Kolmogorov-Smirnov test of normality. Just like in study 1, power was found to be normally distributed in both hemispheres and conditions ($p > .05$), whereas proportion of alpha bursts and duration did not follow a normal distribution ($p < .05$). Since not all the variables were normally distributed, we tested the main effects (contrasts between Lower and Higher Semantic distances) presented on Figure 3, using the non-parametric Wilcoxon test separate per condition (goal-directed vs. free association). Aligned with our hypothesis and with what we found using parametric contrasts (Fig.2), we observed that alpha oscillatory activity was higher in the right temporal area during the generation of goal-directed associations with higher compared to lower semantic distances, in all the three measures, including alpha power ($Z = 3.036$, $p = .002$), duration of alpha bursts ($Z = 2.582$, $p = .010$), and proportion of alpha oscillatory activity ($Z = 2.206$, $p = .027$). These differences were not statistically significant in the free-association condition in any of the measures, including alpha power ($Z =1.192$, $p = .233$), duration ($Z = .623$, $p = .534$), and proportion of alpha activity ($Z =.217$, $p = .828$). On the left hemisphere, the only significant difference between higher and lower semantic distance items was in alpha power, which was higher during goal-directed generation of associations with higher vs. lower semantic distances ($Z = 2.177$, $p = .029$). All the other differences were not statistically significant ($p >.05$). These data can be visualised in Fig.S2.

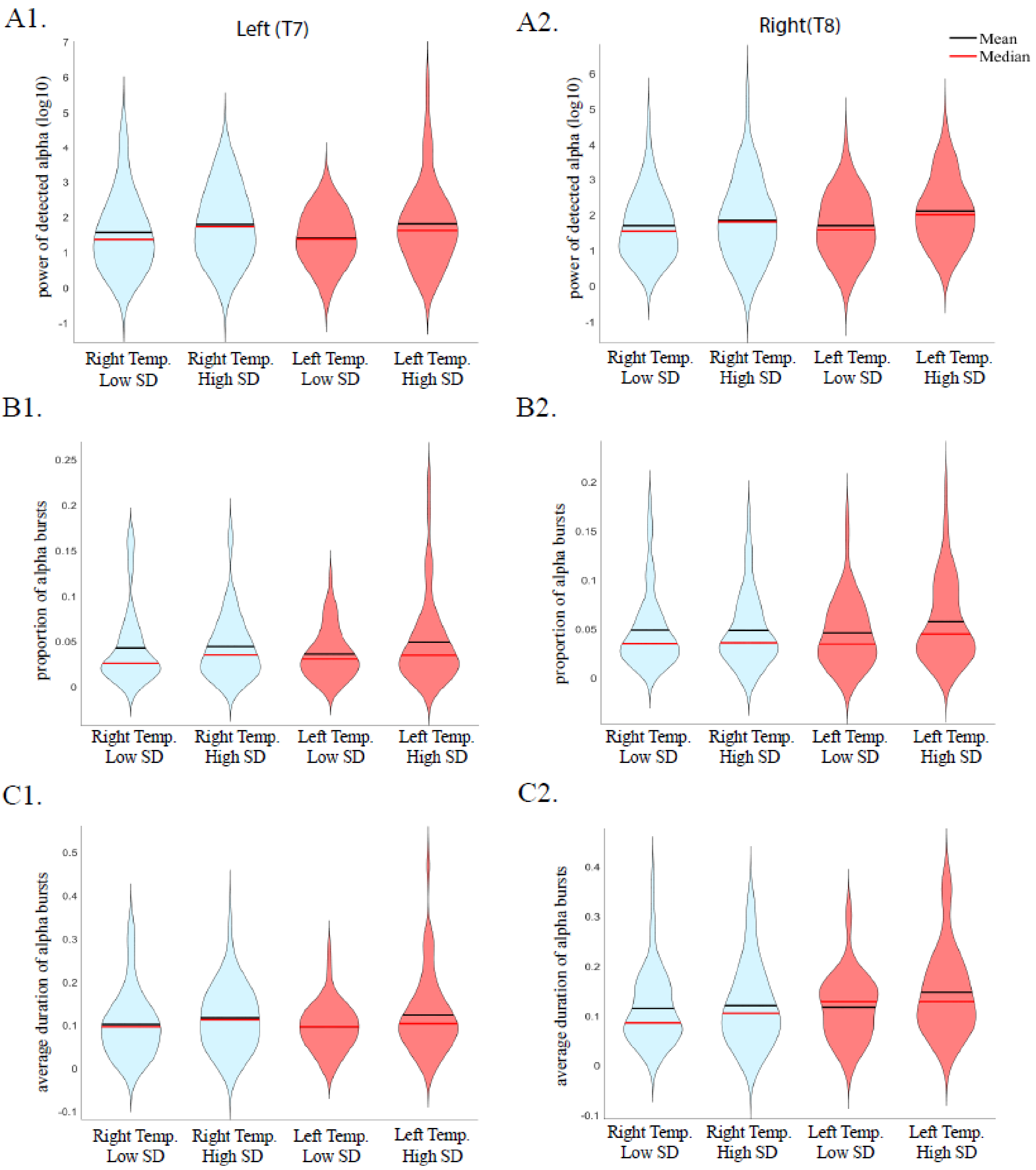

Fig.S2. Violin plots demonstrating the distribution of the three dependent variables in each condition (free association, goal-directed), including: A1. Power of detected alpha oscillations (log10 transformed); B1. Proportion of alpha bursts; C1. Average duration of alpha bursts. A2, B2, and C2 are the same measures but in right hemisphere. Mean is represented with a black line, and median with a red line.

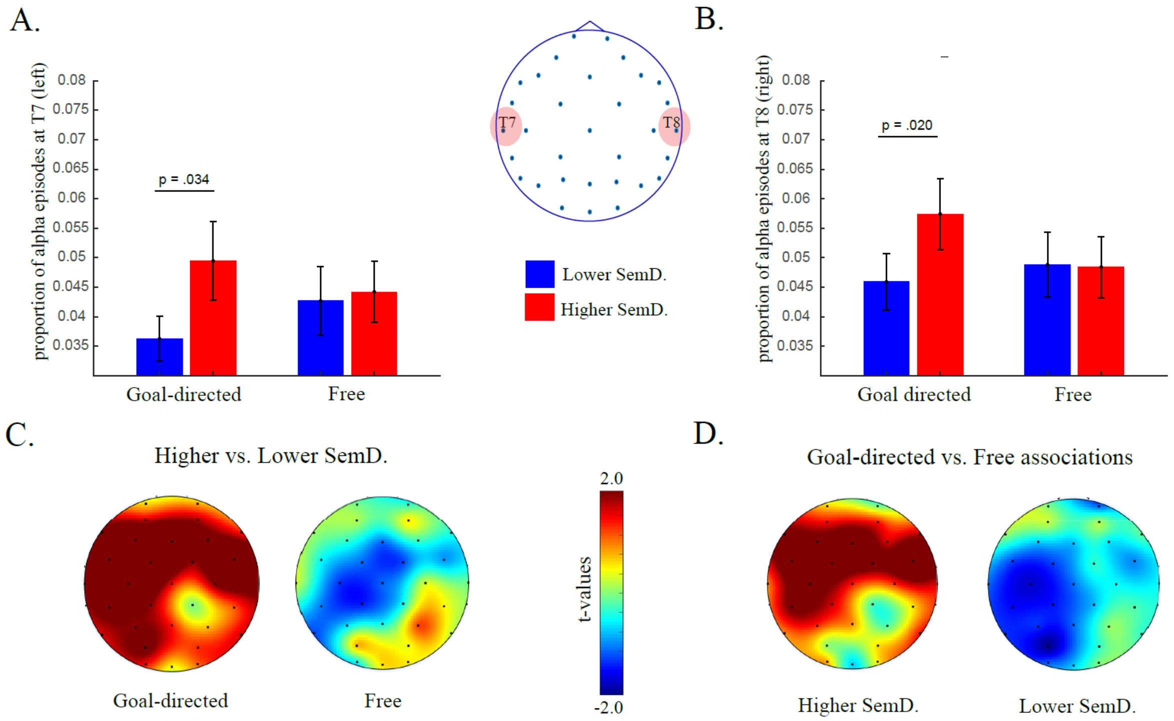

Figure S3: Mean proportion of detected alpha oscillations during the generation of semantic associations. **A/B.** Mean pproportion of detected alpha oscillations during the generation of associations with lower (below median, blue) and higher (above median, red) semantic distance during goal-directed and free association conditions. **A.** in the left temporal area (electrode T7); **B.** in the right temporal area (electrode T8). **C.** Topography of the differences in the proportion of detected alpha oscillations during the generation of higher vs. lower semantic distance. Red-colours indicate larger proportion of alpha oscillatory activity during the generation of items with higher semantic distance compared to lower in the goal-directed (left) and free-association (right) conditions. **D.** Topography of the differences in proportion of detected alpha oscillations during the generation of word associations in the goal-directed vs. free association conditions for associations with lower (left) and higher (right) semantic distance. Red-colours show larger proportion of alpha during goal-directed compared to free generation of associations. The colourbar applies to both C and D topomaps and represents the statistical contrast between the conditions (t-values). Error bars represent +/- 1 S.E.Ms.

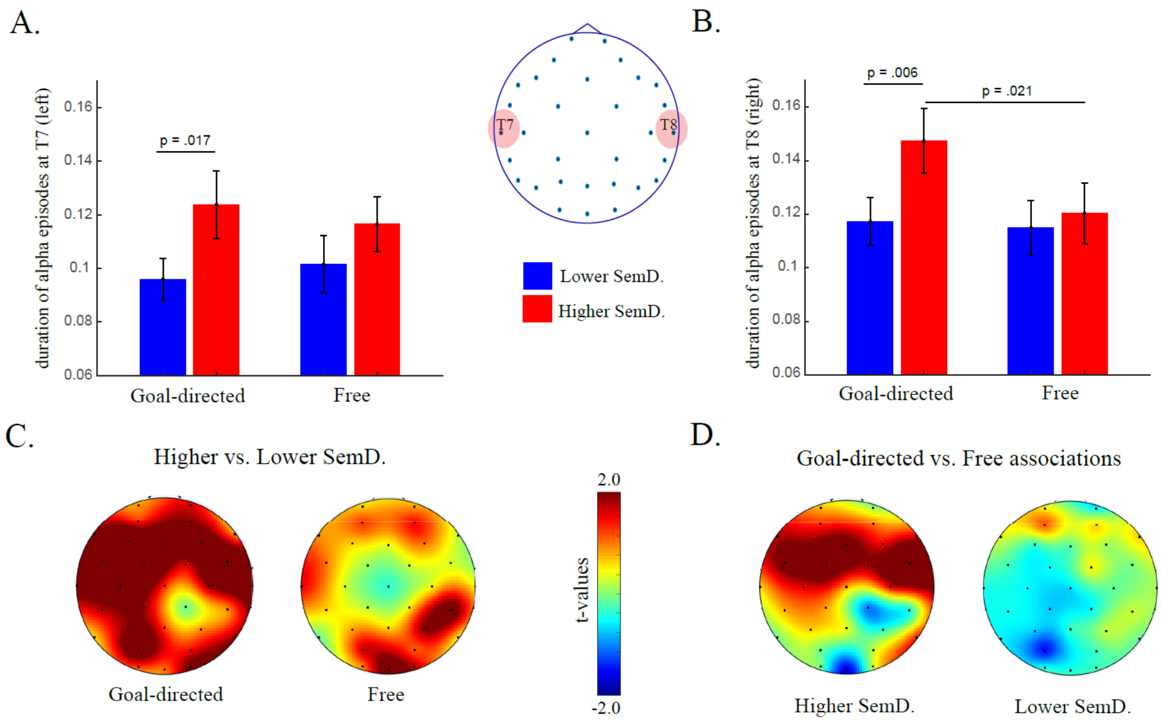

Figure S4: Average duration of alpha oscillatory bursts during the generation of semantic associations. **A/B.** Average duration of alpha oscillatory bursts during the generation of associations with lower (below median, blue) and higher (above median, red) semantic distance during goal-directed and free conditions. **A.** in the left temporal area (electrode T7); **B.** in the right temporal area (electrode T8). **C.** Topography of the differences in the average duration of alpha oscillatory bursts during the generation of higher vs. lower semantic distance. Red-colours indicate larger proportion of alpha oscillatory activity during the generation of items with higher semantic distance compared to lower in the goal-directed (left) and free (right) conditions. **D.** Topography of the differences in average duration of alpha oscillatory bursts during the generation of word associations in the goal-directed vs. free conditions for associations with lower (left) and higher (right) semantic distance. Red-colours show larger average duration of alpha oscillatory bursts during goal-directed compared to free generation of associations. The colourbar applies to both C and D topomaps and represents the statistical contrast between the conditions (t-values). Error bars represent +/- 1 S.E.Ms.

S5: Controlling for order of conditions in Study 2

Considering that in the second study we had participants doing both goal-directed and free associations in the same session, we analysed the potential effects of order. Half of the participants tested did the free associations first whereas the other half did the goal-directed associations first. We tested the effects of order in the behaviour (semantic distances and response times) and in the EEG data analysis (alpha and directed phase synchronization). We did all these analyses using the same dataset we used for all the study 2 analyses described. From these participants, 24 did the free associations first whereas 26 did the goal-directed associations first.

To test the effects of order on semantic distance, we conducted a 2 (*semantic distance: higher and lower)* x 2 (creative intention: free- vs. goal-directed) x 2 (order: free vs. goal-directed first) mixed-design ANOVA. We observed no main effect of order, $F(1,48) = .146$, $p = .704$, *partial* $\eta^2 = .003$, neither interaction between order and semantic distance, $F(1,48) = .339$, $p = .563$, *partial* $\eta^2 = .007$, nor creative intention, $F(1,48) = 1.346$, $p = .252$, *partial* $\eta^2 = .027$. The main effects of semantic distance, $F(1,48) = 2277$, $p < .001$, *partial* $\eta^2 = .979$, and creative intention, $F(1,48) = 4.158$, $p = .047$, *partial* $\eta^2 = .080$, remained similar to the analysis without order as a factor. We conducted the same analysis using response time as dependent variable and observed no main effect of order, $F(1,48) = .232$, $p = .632$, *partial* $\eta^2 = .005$, and interaction between order and semantic distance, $F(1,48) = .088$, $p = .767$, *partial* $\eta^2 = .002$, and between order and creative intention, $F(1,48) = 1.251$, $p = .269$, *partial* $\eta^2 = .025$.

We also considered the possibility of an order effect in the alpha power data. In order to account for that, we repeated the same MANOVA by adding order as a factor. Therefore, we conducted a 2 (semantic distance: lower vs. higher) x 2 (creative intention: free- vs. goal-directed) x 2 (hemisphere: right vs. left) x 2 (order: goal-directed first vs. free-association first) mixed-design MANOVA using alpha power, proportion of alpha oscillatory activity and average alpha burst duration as the dependent variables. We observed no significant main effect of order, $F(3,46) = 1.969$, $p = .132$, *partial* $\eta^2 = .014$, and that order did not interact with any of the factors, including semantic distance, $F(3,46) = .556$, $p = .647$, *partial* $\eta^2 = .035$, creative intention, $F(3,46) = 1.355$, $p = .268$, *partial* $\eta^2 = .081$, and hemisphere, $F(3,46) = .349$, $p = .104$, *partial* $\eta^2 = .124$. There was no significant interaction with order ($p > .05$).

The effects of the main analysis remained the same, with a significant main effect of semantic distance, $F(3,47) = 6.293$, $p = .001$, *partial* $\eta^2 = .291$.

Last, we checked other the effects of creative intention on directed alpha synchronization interacted with order. We conducted a mixed factor 2 (creative intention: free- vs. goal-directed) x 2 (order: goal-directed first vs. free-association first) mixed-design ANOVA on the average cluster PSI values (for the main cluster, from left to right connections displayed in Fig.4B). As expected, we observed a significant main effect of creative intention, $F(1,48) = 24.293$, $p < .001$, *partial* $\eta^2 = .336$. We observed no main effect of order, $F(1,48) = .872$, $p = .355$ *partial* $\eta^2 = .018$, and a marginally non-significant interaction between creative intention and order, $F(1,48) = 3.799$, $p = .057$ *partial* $\eta^2 = .073$. This could have been due to slightly higher mean cluster PSI for goal-directed for when participants started with free associations (mean = .0957, SD = .11) compared to when they started with goal-directed (mean = .0737, SD = .11). However, further contrasts revealed that the difference in cluster PSI between goal-directed and free association conditions is significant for both orders, including starting with the free associations, $t(23) = 4.640$, $p < .001$, *Cohen's D = .947*, and with goal-directed $t(25) = 2.207$, $p = .037$, *Cohen's D = .433.*
